# Intellectual Characteristics in Children With Congenital Unilateral Upper Limb Deficiencies

**DOI:** 10.7759/cureus.37100

**Published:** 2023-04-04

**Authors:** Hiroshi Mano, Sayaka Fujiwara, Chika Nishizaka, Nobuhiko Haga

**Affiliations:** 1 Rehabilitation Medicine, The University of Tokyo Hospital, Tokyo, JPN; 2 Rehabilitation Medicine, Shizuoka Children's Hospital, Shizuoka, JPN

**Keywords:** upper limb function, motor skill, intelligence, congenital limb deformities, adaptive behaviour

## Abstract

Introduction

Some children with motor disabilities show low cognitive levels. However, the influence of motor disabilities on children’s intelligence remains to be fully elucidated. This study aimed to clarify the intellectual characteristics of children with upper limb deficiencies and the influence of upper limb impairments on intelligence.

Methods

The participants were 10 children from four to six years of age with congenital unilateral transradial or transcarpal limb deficiencies who received prosthetic interventions. The children’s intelligence and adaptive behaviors, including motor skills, were examined using the Wechsler Preschool and Primary Scale of Intelligence and the Vineland Adaptive Behavior Scale, respectively.

Results

There were no significant characteristics or discrepancies in cognitive level in children with upper limb deficiencies. The Adaptive Behavior Composite Score of the Vineland Adaptive Behavior Scale was significantly positively correlated with the Full-Scale Intelligence Quotient of the Wechsler Preschool and Primary Scale of Intelligence.

Conclusions

The children with congenital limb deficiencies showed average cognitive levels. Expansion of adaptive behaviors, including appropriate complementation of disabilities, may promote intellectual development in children with motor disabilities.

## Introduction

The incidence of congenital upper limb deficiencies is reportedly 3.4-6.2 per 10,000 total births [1-3]. In Japan, the estimated incidence was 3.39 per 10,000 live births in 2014-2015 [4]. Orthopedic or plastic surgeries and prosthetic or orthotic interventions are treatment options for these patients. Especially for transverse deficiencies, prosthetic interventions could be considered similar to acquired amputations. Children with congenital unilateral transverse upper limb deficiencies are often prescribed prostheses; most use them [5,6].

Children with upper limb deficiencies have disadvantages in motor function. In terms of fine motor skills, cutting paper with scissors, tying a bowknot, zipping up a jacket, and putting up clothes pegs are examples of actions that can be difficult [7]. Children with unilateral transcarpal or transradial upper limb deficiencies who lack adequate experience with prosthetic intervention display weakness in motor skills in the Vineland Adaptive Behavior Scales, Second Edition (Vineland-II) [8,9], a standardized scale of adaptive behavior [10]. Although motor skill weakness increases with age, it can be improved through rehabilitation approaches, including occupational and prosthetic therapies [11]. However, the characteristics of intellectual ability and cognitive functioning and the effect of prostheses or rehabilitation approaches on the intellectual development of these children are unknown.

Some children with motor functional impairments show low cognitive levels. The Wechsler Scales are intelligence tests commonly used worldwide. The Wechsler Intelligence Scale for Children-Fourth Edition (WISC-IV) [12-14] provides the Full-Scale Intelligence Quotient (FSIQ) and four index scores: Verbal Comprehension Index (VCI), Perceptual Reasoning Index (PRI), Working Memory Index (WMI), and Processing Speed Index (PSI). The General Ability Index (GAI), calculated from the VCI and PRI scores, provides a summary score that is less sensitive to the influence of working memory and processing speed than that provided by the FSIQ. The Cognitive Proficiency Index (CPI), calculated from the WMI and PSI scores, represents a set of functions whose common element is the proficiency with which a person processes certain types of cognitive information.

According to the WISC-IV manuals, children with motor deficits demonstrate low FSIQ, PRI, and PSI scores, and it was suggested that the VCI or GAI were more accurate indices of intrinsic comprehension in those children [12,14]. For example, children with myelomeningocele without delays in intellectual development showed average GAI scores and low PSI and CPI scores [15]. The Wechsler Preschool and Primary Scale of Intelligence-Third Edition (WPPSI-III) [16,17] provides the FSIQ, three index scores (VCI, PRI, and PSI), and one optional index score of the general language composite (GLC). According to the WPPSI-III manual, children with motor deficits demonstrated low FSIQ, PRI, and PSI scores, and it was suggested that the VCI was a more accurate index of intrinsic comprehension in those children [16]. However, motor functional impairments in children are often the result of central nervous system disorders, such as cerebral palsy and spina bifida. The sole effect of motor disabilities on children’s intelligence still needs to be elucidated. This study aimed to clarify the intellectual characteristics of children with upper limb deficiencies and the effect of upper limb disabilities on intelligence.

## Materials and methods

This study was approved by the Ethics Committee of the Faculty of Medicine of the University of Tokyo (approval number 10706). The participants were children aged four to six years with congenital unilateral transradial or transcarpal limb deficiency recruited from the outpatients visiting our university hospital. This study was carried out at Rehabilitation Medicine, The University of Tokyo Hospital, Tokyo, Japan. Those diagnosed with coexisting disorders affecting intelligence or adaptive behaviors, such as autism spectrum disorder, attention deficit hyperactivity disorder, or intellectual disability, were excluded. The study’s aims and methods were explained to the participating children and their guardians. Oral informed consent was obtained from the participating children, and written informed consent was obtained from their guardians.

We examined the children’s intellect using the WPPSI-III and evaluated adaptive behaviors, including motor skills, using the Vineland-II. At the time of the WPPSI-III test, the participants were free to choose which, if any, prosthesis to wear to facilitate desk tasks. All tasks were performed with the same prosthesis if one was chosen. The scaled scores of the subtests (standard score with a mean of 10 and a standard deviation of 3), the FSIQ (standard score with a mean of 100 and a standard deviation of 15), and the index scores (standard score with a mean of 100 and a standard deviation of 15) were calculated. From these scores, the children’s intellectual characteristics were evaluated. In the present study, we performed four subtests for VCI, four for PRI, and two for PSI. We did not perform any subtests for GLC.

The Vineland-II scale uses standard scores to describe an individual’s overall functioning and level of function within each adaptive behavior domain. Domains and adaptive behavior composite standard scores have means of 100 and standard deviations of 15. The v-scale scores, which describe the individuals’ relative functioning level compared to others of the same age within the subdomains, have a mean of 15 and standard deviations of 3. The present study assessed all adaptive behavior domains (communication, daily living skills, socialization, and motor skills) and subdomains. We did not perform an optional maladaptive domain.

Each standard score of the WPPSI-III was analyzed using a t-test under the hypothesis of a mean of 10 for subtest scaled scores and a mean of 100 for the FSIQ and index scores. To compare the index scores and subtest scores in the WPPSI-III, a paired t-test was used. To compare the WPPSI-III and the Vineland-II scores, Pearson product-moment correlation coefficients were calculated. JMP® 14 (SAS Institute Inc., Cary, NC, USA) was used for all statistical analyses. P-values < 0.05 were considered statistically significant.

## Results

Participants

The participants’ characteristics, including age, sex, level of deficiency, prostheses used, and Vineland-II scores, are summarized in Table 1 and Table 2. The standard occupational therapy courses and prosthetic interventions for children with upper limb deficiencies at our hospital are indicated below. Initially, passive hand prostheses were prescribed, and occupational therapy was initiated. After six months to one year, when the children and their guardians became familiar with their prostheses, prescriptions for prostheses with voluntary hands, such as myoelectric prostheses, were considered, depending on the child’s needs. Occupational therapy included training regarding prosthesis usage and compensatory movements without using prostheses. All children in the present study followed the standard occupational therapy course, as described above. During the WPPSI-III test, two children (four and nine) chose to use passive hand prostheses, and eight chose not to use their prostheses to perform the tasks. None chose to use myoelectric prostheses.

**Table 1:** Characteristics of the participants in the present study † WPPSI-III: Wechsler Preschool and Primary Scale of Intelligence-Third Edition

Child	Age (years)	Sex	Level of deficiency	Use of prosthesis			Prosthetic use on the occasion of WPPSI-III† test
				Duration (years)	Passive hand	Myoelectric hand	
1	6.6	Male	partial carpal	3.2	past use	in use	no use
2	6.6	Male	complete carpal	3.6	past use	in use	no use
3	5.5	Female	partial carpal	0.3	in use		no use
4	5.3	Female	partial carpal	4.0	in use	in use	passive hand
5	5.2	Male	forearm	3.9	in use	in use	no use
6	4.5	Male	partial carpal	2.3	past use	in use	no use
7	4.4	Male	partial carpal	2.4	in use	in use	no use
8	4.3	Female	partial carpal	1.5	past use	in use	no use
9	4.1	Female	forearm	2.8	in use	in use	passive hand
10	4.1	Female	forearm	2.5	in use	in use	no use

**Table 2:** Adaptive Behavior Composite Score, domain standard scores, and subdomain V-scale scores using Vineland-II† † Vineland-II: Vineland Adaptive Behavior Scales, Second Edition ‡ The standard scores have a mean of 100 and a standard deviation of 15. § The standard scores have a mean of 15 and a standard deviation of 3.

Domains and Subdomains	Mean	Standard Deviation
Adaptive behavior composite^‡^	103.6	3.8
Communication^‡^	101.3	7.5
Receptive^§^	15.8	1.2
Expressive^§^	15.1	1.3
Written^§^	15.2	1.0
Daily Living Skills^‡^	104.9	7.8
Personal^§^	15.4	2.0
Domestic^§^	16.4	1.6
Community^§^	16.4	0.8
Socialization^‡^	99.4	5.3
Interpersonal Relationships^§^	13.8	0.6
Play and Leisure Time^§^	17.3	1.3
Coping Skills^§^	14.3	1.5
Motor Skills^‡^	100.9	6.8
Gross^§^	14.7	1.4
Fine^§^	15.7	1.2

WPPSI-III

Table 3 shows each of the standard scores of the WPPSI-III. All the mean values of the FSIQ, index scores, and subtest scores were averaged. There were no significant differences between the mean values of children with motor impairments and the general population.

**Table 3:** Full-Scale Intelligence Quotient, index scores, and subtest scale scores of WPPSI-III† † WPPSI-III: Wechsler Preschool and Primary Scale of Intelligence-Third Edition; FSIQ: Full-Scale Intelligence Quotient ‡ The FSIQ and the index scores have a mean of 100 and a standard deviation of 15. §  The subtest scores have a mean of 10 and a standard deviation of 3. ¶  t-test

Indexes and Subtests	Mean	Standard Deviation	p-Value^¶^
Full Scale Intelligence Quotient (FSIQ)^‡^	99.7	17.5	0.96
Verbal Comprehension Index (VCI)^‡^	102.8	18.2	0.64
Information^§^	9.9	3.8	0.94
Vocabulary^§^	10.6	4.0	0.65
Word reasoning^§^	9.3	2.9	0.47
Comprehension^§^	10.2	3.2	0.85
Perceptual Reasoning Index (PRI)^‡^	97.1	13.4	0.51
Block design^§^	8.5	2.5	0.09
Matrix reasoning^§^	9.7	3.5	0.80
Picture Concepts^§^	9.7	2.1	0.66
Picture Completion^§^	9.8	2.9	0.83
Processing Speed Index (PSI)^‡^	97.1	16.6	0.59
Symbol Search^§^	8.9	2.2	0.15
Coding^§^	9.8	3.4	0.86

Table 4 shows the differences between the WPPSI-III scores. There were no significant differences between index or subtest scores.

**Table 4:** Pairwise comparisons of scores of the WPPSI-III† † WPPSI-III: Wechsler Preschool and Primary Scale of Intelligence-Third Edition; FSIQ: Full-Scale Intelligence Quotient; VCI: Verbal Comprehension Index; PRI: Perceptual Reasoning Index; PSI: Processing Speed Index ‡ paired t-test

Indexes and Subtests	Mean	Standard Error of the Mean	p-Value^‡^
Index scores			
VCI -PRI	+5.7	4.6	0.25
VCI - PSI	+5.7	5.2	0.30
PRI - PSI	0.0	5.9	1.00
Subtest scores of VCI			
Information - Vocabulary	-0.7	0.8	0.42
Information - Word reasoning	+0.6	1.1	0.60
Information - Comprehension	-0.3	1.1	0.79
Vocabulary - Word reasoning	+1.3	1.0	0.24
Vocabulary - Comprehension	+0.4	0.9	0.67
Word reasoning - Comprehension	-0.9	0.6	0.16
Subtest scores of PRI			
Block design - Matrix reasoning	-1.2	0.9	0.22
Block design - Picture Concepts	-1.2	0.8	0.17
Block design - Picture Completion	-1.3	1.0	0.20
Matrix reasoning - Picture Concepts	0.0	1.2	1.00
Matrix reasoning - Picture Completion	-0.1	0.8	0.91
Picture Concepts - Picture Completion	-0.1	1.2	0.94
Subtest scores of PSI			
Symbol Search - Coding	-0.9	0.55	0.13

Table 5 shows the relationship between the composite WPPSI-III and Vineland-II scores. The Adaptive Behavior Composite Score was significantly positively correlated with the FSIQ and PRI scores. However, motor skill scores did not correlate with the WPPSI-III composite scores.

**Table 5:** Pearson product-moment correlation coefficient between the composite scores of the WPPSI-III† and the Vineland-II‡ † WPPSI-III: Wechsler Preschool and Primary Scale of Intelligence-Third Edition ‡ Vineland-II: Vineland Adaptive Behavior Scales, Second Edition

Domains/Indexes	Full Scale Intelligence Quotient (FSIQ)		Verbal Comprehension Index (VCI)		Perceptual Reasoning Index (PRI)		Processing Speed Index (PSI)	
	Coefficient of correlation	p-value	Coefficient of correlation	p-value	Coefficient of correlation	p-value	Coefficient of correlation	p-value
Adaptive behabior composite	0.65	0.04	0.52	0.13	0.66	0.04	0.38	0.28
Communication	0.59	0.07	0.51	0.13	0.39	0.27	0.62	0.054
Daily Living Skills	0.27	0.45	0.11	0.77	0.36	0.30	0.27	0.45
Socialization	0.24	0.50	0.15	0.68	0.28	0.43	0.26	0.46
Motor Skills	0.05	0.88	0.16	0.66	0.14	0.70	-0.48	0.16
Gross	-0.08	0.83	-0.11	0.77	0.14	0.70	-0.40	0.26
Fine	0.22	0.54	0.42	0.23	0.09	0.81	-0.28	0.44

## Discussion

In this study, children with congenital upper limb deficiencies did not have any significant characteristics or discrepancies in comprehension; that is, they showed average comprehension in the WPPSI-III. There were no correlations between motor skills in the Vineland-II and the WPPSI-III scores. Although children with motor functional impairments show low intelligence in Wechsler tests [12,14,16], our results contradict this. There are two possible causes for this: one is that the impairment of the unilateral hand may have a little negative effect on intellectual development. Another is that although the impairment may affect intellectual development, appropriate complementation of upper limb function with prosthetic interventions and occupational therapy may promote intellectual development. If the latter is assumed to be accurate, to enhance intellectual development in children with motor disabilities, their motor functions should be appropriately complemented as much as possible.

In this study, children with congenital upper limb deficiencies showed average scores on the Vineland-II scale, including motor skills scores. The Vineland-II manual stated that the Adaptive Behavior Composite Score of the Vineland-II positively and strongly correlated with the intelligence quotient of the Wechsler or Binet tests [8,9]. The Adaptive Behavior Composite Score significantly correlated with the FSIQ and PRI in this study. However, no Vineland-II domain standard scores, including that of motor skills, correlated with the FSIQ and WPPSI-III index scores. We surmised that it is necessary to provide appropriate treatments and support to children with disabilities from an early age without limiting specific skills. Complementation of motor function, improvement of activities of daily living, and expansion of social participation may be important for developing intellect in children with congenital upper limb deficiencies.

There was no correlation between the motor skills scores of the Vineland-II and the WPPSI-III subtest scores. At the time of the WPPSI-III test, only two children chose to use passive hand prostheses, and eight chose not to use their prostheses to perform the tasks. No children chose to use a myoelectric prosthesis. Unilateral transradial or transcarpal deficiencies had minimal effects on the performance of the comprehension tests. A disadvantage of myoelectric prosthetic hands is that they take a little time to open and close, which can sometimes be disadvantageous for desk tasks. Furthermore, artificial hands have no sensory feedback, such as tactile sensation. However, this does not mean that these prostheses are useless for intellectual tasks. Even if the children completed movements without their prostheses, there were cases in which prosthetic intervention played an important role. For example, most children in our hospital first learned to tie a secure bow with a prosthesis, and later, some children learned to tie a secure bow without a prosthesis. Although children may eventually learn to tie secure bows even if they never use prostheses, they can more easily understand by using prostheses and moving both hands. Complementing cosmetic and motor functions with prostheses and occupational therapy may encourage children and their guardians to participate in society and acquire various experiences. The experience of holding or lifting an object with both hands, looking at it from various angles, and performing activities through individual practice promotes deep understanding. Prosthetic interventions, including occupational therapy, may provide such opportunities to children with motor disabilities.

Limitations

Due to the rarity of the studied disorder, only 10 children were included in this study. The small sample size may have influenced the results. Furthermore, we clarified the intellectual characteristics of preschool children with congenital limb deficiencies. These characteristics in school-age children and adults are still unknown. In addition, the intellectual characteristics of children who have never received prosthetic interventions or occupational therapy are still unknown. As prosthetic intervention and occupational therapy begin early in infancy with a general course of treatment, it is difficult to measure comprehension in the untreated or pre-treatment condition because there are no appropriate intelligence tests that can be administered early in infancy. Confounding factors, such as socioeconomic status, that might affect the results were not controlled for in this study. Thus, further research examining other developmental stages with larger sample sizes will be needed to resolve such issues.

## Conclusions

This study investigated the cognitive levels of children with congenital limb deficiencies who received prosthetic interventions. The intelligence of children with upper limb deficiencies did not have any distinguishing characteristics or discrepancies; that is, they showed average comprehension. There are two possible causes for this: one is that unilateral hand disabilities may have a little negative effect on intellectual development. Another is that although the disabilities may affect intellectual development, appropriate complementation of upper limb function may promote it. Comprehension was significantly positively correlated with adaptive behavior. Expansion of adaptive behaviors, including appropriate complementation of disabilities, may promote intellectual development in children with motor disabilities.

## References

[1] Bedard T, Lowry RB, Sibbald B, Kiefer GN, Metcalfe A (2015). Congenital limb deficiencies in Alberta-a review of 33 years (1980-2012) from the Alberta congenital anomalies surveillance system (ACASS). Am J Med Genet A.

[2] Vasluian E, van der Sluis CK, van Essen AJ, Bergman JE, Dijkstra PU, Reinders-Messelink HA, de Walle HE (2013). Birth prevalence for congenital limb defects in the northern Netherlands: a 30-year population-based study. BMC Musculoskelet Disord.

[3] Castilla EE, Cavalcanti DP, Dutra MG, Lopez-Camelo JS, Paz JE, Gadow EC (1995). Limb reduction defects in South America. Br J Obstet Gynaecol.

[4] Mano H, Fujiwara S, Takamura K (2018). Congenital limb deficiency in Japan: a cross-sectional nationwide survey on its epidemiology. BMC Musculoskelet Disord.

[5] Kuyper MA, Breedijk M, Mulders AH, Post MW, Prevo AJ (2001). Prosthetic management of children in the Netherlands with upper limb deficiencies. Prosthet Orthot Int.

[6] Mano H, Fujiwara S, Takamura K, Kitoh H, Takayama S, Ogata T, Haga N (2021). Treatment approaches for congenital transverse limb deficiency: data analysis from an epidemiological national survey in Japan. J Orthop Sci.

[7] Mano H, Noguchi S, Fujiwara S, Haga N (2021). Relationship between degree of disability, usefulness of assistive devices, and daily use duration: an investigation in children with congenital upper limb deficiencies who use upper limb prostheses. Assist Technol.

[8] Tsujii M, Murakami T, Kuroda M, Ito H, Hagiwara T, Someki F (2014). Japanese Version of the Vineland Adaptive Behavior Scales, Second Edition (Book in Japanese). https://www.nichibun.co.jp/seek/kensa/vineland2.html.

[9] Sparrow SS, Cicchetti DV, Balla DA, Doll EA (2005). Vineland Adaptive Behavior Scales, Second Edition: Survey Forms Manual. https://pearsonclinical.in/solutions/vineland-ii/.

[10] Mano H, Fujiwara S, Haga N (2018). Adaptive behaviour and motor skills in children with upper limb deficiency. Prosthet Orthot Int.

[11] Mano H, Fujiwara S, Haga N (2020). Effect of prostheses on children with congenital upper limb deficiencies. Pediatr Int.

[12] Wechsler D (2010). Technical and Interpretive Manual for the Japanese Version of the Wechsler Intelligence Scale for Children-Fourth Edition (Book in Japanese). https://www.nichibun.co.jp/seek/kensa/wisc4.html.

[13] Wechsler D (2010). Administration and Scoring Manual for the Japanese Version of the Wechsler Intelligence Scale for Children-Fourth Edition (Book in Japanese).

[14] Wechsler D (2014). Administration and Scoring Manual for the Japanese Version of the Wechsler Intelligence Scale for Children-Fourth Edition (Supplemental Manual).

[15] Mano H, Takikawa K, Haga N (2018). Intellectual characteristics using WISC-IV in children with myelomeningocele. Cogent Medicine.

[16] Wechsler D (2017). Technical and Interpretive Manual for the Japanese Version of the Wechsler Preschool and Primary Scale of Intelligence-Third Edition (Book in Japanese). https://www.nichibun.co.jp/seek/kensa/wppsi3.html.

[17] Wechsler D (2017). Administration and Scoring Manual for the Japanese Version of the Wechsler Preschool and Primary Scale of Intelligence-Third Edition (Book in Japanese).

